# Supplementary material for: The Role and Mechanism of GSDME-Dependent Pyroptosis in Cochlear Marginal Cells Injury by Cisplatin
Source: Biomedicines. 2025 Jul 9;13(7):1680. doi: 10.3390/biomedicines13071680 (PMC12292857; doi:10.3390/biomedicines13071680)
Supplement: Supplementary file 1 [file biomedicines-13-01680-s001.zip › biomedicines-3696009-supplementary.pdf]

Supplementary Table S1. The primer sequences used in real-time quantitative PCR

Genes	Forward Primer	Reverse Primer
**GAPDH**	GAAGGTCGGTGTGAACGGAT	CCCATTTGATGTTAGCGGGAT
**GSDME**	TATTTGGAAGTGGCCTGCGT	CTCCTCTCTAGGTGCAGGGT
**Caspase-3**	GCAGCTAACCTCAGAGAGACATT	CAGTAGTCGCCTCTGAAGAAACT

Supplementary Table S2. The primer sequences of six candidate reference genes used in real-time quantitative PCR

Genes	Forward Primer	Reverse Primer
**GAPDH**	GAAGGTCGGTGTGAACGGAT	CCCATTTGATGTTAGCGGGAT
**ACTB**	GGACCTGACAGACTACCTCA	GTTGCCAATAGTGATGACCT
**HPRT1**	TAGCACCTCCTCCGCCAG	CACTAATCACGACGCTGGGA
**B2M**	GCAAGGACTGGTCTTTCTAC	CAGATGATTCAGAGCTCCATAG
**RPLP0**	CCCTTCTCCTTCGGGCTGAT	TGAGGCAACAGTCGGGTAGC
**SDHA**	CTACTGTACCTCCTGCTATCC	CTGTGAAGTGACTCCTTGTTC

GAPDH, glyceraldehyde 3-phosphate dehydrogenase; ACTB, actin beta;HPRT, hypoxanthine phosphoribosyltransferase; B2M, beta-2-microglobulin; RPLP0, ribosomal protein lateral stalk subunit P0; SDHA, succinate dehydrogenase complex flavoprotein subunit A.

Supplementary Table S3. The main experimental equipment and suppliers used in this study.

experimental equipment	Suppliers
Horizontal gel electrophoresis system	Servicebio Co., Ltd (Wuhan, China)
Inverted microscope	Olympus Corporation, Japan
Ultracentrifuge (low-temperature)	Jiangsu Tianli Medical Equipment Co., Ltd, China
Portable autoclave	Ningbo Jiuxing Medical Instrument Co., Ltd, China
NanoDrop spectrophotometer	Bio-Rad Laboratories, USA
Electroblotting transfer system	Thermo Fisher Scientific, USA
Water-jacketed CO2 incubator	Shanghai Yuejin Medical Equipment Co., Ltd, China
Laser scanning confocal microscope	Nikon Instruments, Japan
Real-time PCR system	Roche Diagnostics, USA
Ultrapure water purification system	Jinan OLABO Scientific, China

Magnetic stirrer	Jintan Guorui Experimental Equipment, China
Glass plate set (for gel electrophoresis)	Servicebio Co., Ltd (Wuhan, China)
Electrophoresis comb (1.0 mm thickness)	Servicebio Co., Ltd (Wuhan, China)
Integrated horizontal electrophoresis system	Thermo Fisher Scientific, USA
CO_2 incubator (water-jacketed)	Shanghai Yuejin Medical Equipment Co., Ltd, China
Microplate reader (multimode)	BioTek Instruments,USA
Ultrasonic cell disruptor	Sonics & Materials, Inc. USA
Dry bath incubator (metal)	Guangzhou Biolight Biotechnology
Flow cytometer	Thermo Fisher Scientific, USA

Supplementary Table S4. The main reagents and suppliers used in this study.

Reagent Name	Supplier
Absolute ethanol	Sinopharm Chemical Reagent Co., Ltd,China
Hanks' balanced salt solution	Servicebio Biotechnology Co., Ltd,Wuhan, China
Isopropanol	Sinopharm Chemical Reagent Co., Ltd,Guangzhou, China
DAPI	Antgene Biotechnology Co., Ltd ,Wuhan, China
Collagenase Type II	Merck & Co., Inc.,USA
Cisplatin injection	Yunnan Plant Pharmaceutical Co., Ltd,China
RNA Quantification Kit	Vazyme Biotech Co., Ltd,Nanjing, China
Tween20	Biofroxx,Germany
PBS buffer	Servicebio Biotechnology Co., Ltd,Wuhan, China
CCK-8 assay solution	ShuoPu Biotechnology Co., Ltd,China
DEPC-treated water	Sinopharm Chemical Reagent Co., Ltd,China
Reverse transcription kit	Vazyme Biotech Co., Ltd,Nanjing, China
Paraformaldehyde (solid)	Vazyme Biotech Co., Ltd,Nanjing, China
Adhesive microscope slides	Jiangsu Shitai Experimental Equipment Co., Ltd,China
Normal donkey serum	Antgene Biotechnology Co., Ltd,Wuhan, China
Cell culture plates	Servicebio Biotechnology Co., Ltd,Wuhan, China

GSDME antibody(13075-1-AP)	Wuhan Sanying Biotechnology Co., Ltd,China
Alexa Fluor647 Donkey anti Rabbit IgG	Antgene Biotechnology Co., Ltd ,Wuhan, China
Chloroform	Sinopharm Chemical Reagent Co., Ltd,China
EpiCM-A epithelial cell medium	ScienCell Research Laboratories,USA
Sterile cell climbing slides	ShuoPu Biotechnology Co., Ltd,Guangzhou, China
Triton-100	Sinopharm Chemical Reagent Co., Ltd,China
Caspase-3 antibody(19677-1-AP)	Wuhan Sanying Biotechnology Co., Ltd,China

Supplementary Table S5. The RT-PCR method lacks information on the RNA concentration in the reaction mixture and the PCR reaction conditions

Step	Description	Cycles	Time (sec)	Temperature (°C)
**Step**1	Denaturation	1	60	95
**Step**2	Cycling	60	3-10	90
**Step**3	Cycling	60	10-30	60
**Step**4	Melting curve	1	15	95

Supplementary Table S6. A list of all abbreviations used in the text along with their corresponding full terms.

Abbreviation	Full Term
caspase-3	Cysteine-dependent Aspartate-specific Protease-3
CTR1	Copper Transporter 1
DAPI	4',6-diamidino-2-phenylindole
DMEM	Dulbecco's Modified Eagle Medium
cDNA	complementary DNA
ΔΔCT	comparative cycle threshold
EM	electron microscopy
GSDME	gasdermin E
GSDMD	gasdermin D
HRP	horseradish peroxidase
IC50	half-maximal inhibitory concentrations
LDH	lactate dehydrogenase

MCs	marginal cells
OCT2	Organic Cation Transporter 2
PBS	phosphate-buffered saline
PBST	PBS containing 0.1% Tween-20
PVDF	polyvinylidene fluoride
PI	propidium iodide
qRT-PCR	Quantitative real-time polymerase chain reaction
RIPA	Radio-Immunoprecipitation Assay
rpm	revolutions per minute
SD	Sprague-Dawley
SDS-PAGE	SDS-polyacrylamide gel electrophoresis
SEM	scanning electron microscopy
siRNA	small interfering RNA
TEM	transmission electron microscopy

Supplementary Table S7. This study measured both the original and diluted protein concentrations across various sample groups and experimental treatment conditions.

Simple ID	Group	Original conc. (μg/μL)	Final conc. (μ g/ μ L)
1	control	2.9174992	0.6864704
1	5μM cisplatin	2.2986958	0.5408696
2	control	2.004469133	1.145410933
2	5μM cisplatin	2.178806933	1.245032533
3	control	2.828805467	0.808230133
3	5μM cisplatin	2.092712533	0.597917867
4	control	2.811746178	1.533679733
4	5μM cisplatin	1.948500889	1.062818667
5	control	2.5513236	1.4578992
5	5μM cisplatin	2.159436067	1.233963467

6	control	3.198342	0.913812
6	5μM cisplatin	2.620196133	0.748627467

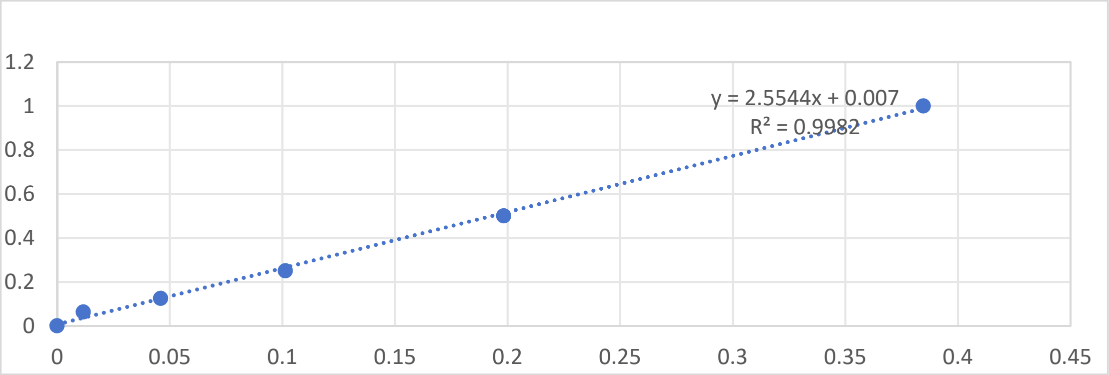

Supplementary Figure S1. Standard calibration curve for protein quantification. The curve was generated by measuring the absorbance of serially diluted standard protein solutions, demonstrating the linear relationship between protein concentration (X-axis) and absorbance signal (Y-axis).

Supplementary Table S8. RNA concentrations in experimental and control groups.

Simple ID	Group	RNA concentration (A260) (ng/ul)
1	Control 1	518. 8
1	Control 2	620. 3
1	Control 3	556. 9
1	5μM cisplatin 1	258. 5
1	5μM cisplatin 2	113. 9
1	5μM cisplatin 3	239. 8
2	Control 1	316. 4
2	Control 2	337
2	Control 3	1451. 8
2	5μM cisplatin 1	648. 3
2	5μM cisplatin 2	24. 6
2	5μM cisplatin 3	234. 5

3	Control 1	213.5
3	Control 2	275.1
3	Control 3	373.1
3	5μM cisplatin 1	64.6
3	5μM cisplatin 2	55.7
3	5μM cisplatin 3	55.2
4	Control 1	563.7
4	Control 2	1486.6
4	Control 3	644.5
4	5μM cisplatin 1	258
4	5μM cisplatin 2	547.9
4	5μM cisplatin 3	286.5
5	Control 1	563.7
5	Control 2	1486.6
5	Control 3	644.5
5	5μM cisplatin 1	258
5	5μM cisplatin 2	547.9
5	5μM cisplatin 3	286.5
6	Control 1	419
6	Control 2	286.5
6	Control 3	269.9
6	5μM cisplatin 1	41
6	5μM cisplatin 2	8.8

6 5μM cisplatin 3

44. 7

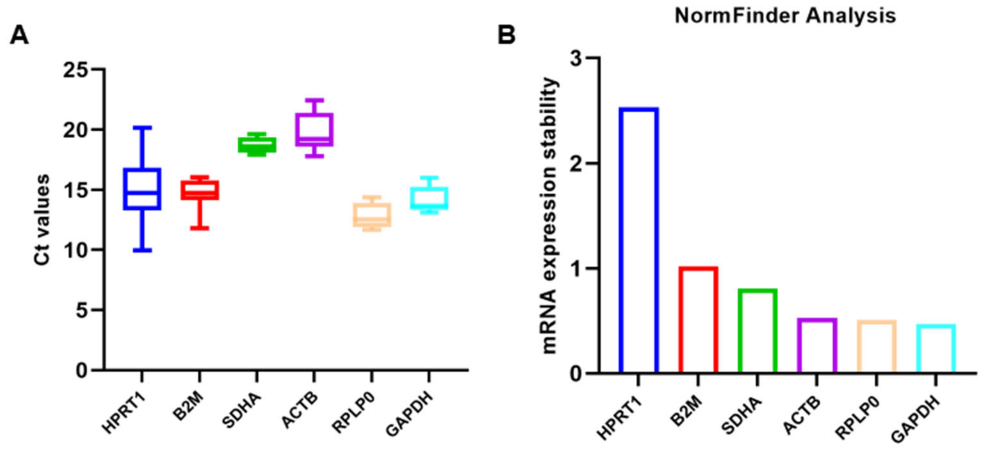

Supplementary Figure S2. The mRNA expression stability analysis results of the six candidate reference genes. A. The mRNA expression stability results of the six candidate reference genes in the lateral wall of the stria vascularis in SD rats; B. The stability of mRNA expression for the six candidate reference genes was evaluated using the NormFinder software. This software calculates a stability value for each gene, with lower values indicating more stable expression.

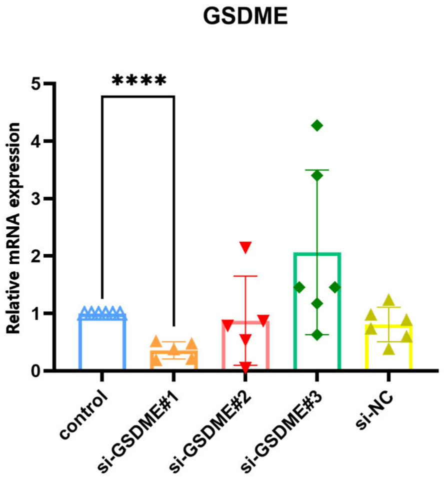

Supplementary Figure S3. Differential expression of GSDME mRNA in marginal cells following small interfering RNA (siRNA) treatment. Transfection of three distinct GSDME-targeting siRNA sequences into marginal cells revealed significant variations in GSDME mRNA expression levels as measured by quantitative PCR. Notably, siRNA-GSDME#1 demonstrated superior interference efficiency, suppressing GSDME expression by >80% in marginal cells, which substantially exceeded the knockdown effects achieved by sequence #2 and sequence #3. All data represent n⩾3 biological replicates (independent animals), with experiments repeated three times independently. Statistical significance was determined by one-way

ANOVA followed by Tukey's HSD post hoc test and is indicated as follows: *p < 0.05, **p < 0.01, ***p < 0.001, ****p < 0.0001, ns: No significant difference.

Supplementary Data S1: CT Values from qPCR Analysis of Six Candidate Reference Genes.

**Housekeeping gene (GAPDH)**		
**simple**	**Cт value**	**Cт mean**
Control	13.83316803	
Control	13.50920486	13.32
Control	12.62360954	
Cisplatin	10.08459568	
Cisplatin	16.66346169	13.10
Cisplatin	12.55605888	
Z-DEVD-FMK	18.68860817	
Z-DEVD-FMK	14.15575695	15.66
Z-DEVD-FMK	14.12456989	
SiRNA-GSDME	13.17271423	
SiRNA-GSDME	13.43966866	13.69
SiRNA-GSDME	14.46789742	
Control	13.83362579	
Control	13.17818451	13.41
Control	13.22526932	
Cisplatin	10.74844551	
Cisplatin	17.54060936	13.64
Cisplatin	12.62199211	
Z-DEVD-FMK	19.01169395	
Z-DEVD-FMK	14.43953896	16.00
Z-DEVD-FMK	14.54429722	
SiRNA-GSDME	12.67723465	
SiRNA-GSDME	13.23507595	13.96
SiRNA-GSDME	15.98134995	

Housekeeping gene (ACTB)		
**simple**	**C_T value**	**C_T mean**
Control	19.8531456	
Control	19.98274994	19.52
Control	18.72156143	
Cisplatin	14.51051903	
Cisplatin	19.90399551	17.78
Cisplatin	18.93109322	
Z-DEVD-FMK	24.31702614	
Z-DEVD-FMK	20.97443581	22.45
Z-DEVD-FMK	22.05635262	
SiRNA-GSDME	20.96575356	
SiRNA-GSDME	18.79309654	19.88
SiRNA-GSDME	Undetermined	
Control	19.45428467	
Control	18.75308228	18.92
Control	18.55021095	
Cisplatin	16.69693184	
Cisplatin	19.90805817	18.83
Cisplatin	19.88997269	
Z-DEVD-FMK	23.94835281	
Z-DEVD-FMK	20.34095764	21.91
Z-DEVD-FMK	21.43247986	
SiRNA-GSDME	19.25839424	
SiRNA-GSDME	19.27350426	18.52
SiRNA-GSDME	17.0399704	

Housekeeping gene (HPRT1)		
**simple**	**Cт value**	**Cт mean**
Control	16.33170891	
Control	16.25438499	17.34
Control	19.43537331	
Cisplatin	9.333381653	
Cisplatin	19.99537468	14.75
Cisplatin	14.90954304	
Z-DEVD-FMK	20.97383881	
Z-DEVD-FMK	19.36341476	20.17
Z-DEVD-FMK	Undetermined	
SiRNA-GSDME	5.164535999	
SiRNA-GSDME	18.33054543	14.70
SiRNA-GSDME	20.60402298	
Control	15.9623661	
Control	18.98404884	12.98
Control	3.984527349	
Cisplatin	Undetermined	
Cisplatin	4.130954266	9.94
Cisplatin	15.7521553	
Z-DEVD-FMK	20.9603157	
Z-DEVD-FMK	19.48525429	15.28
Z-DEVD-FMK	5.395665646	
SiRNA-GSDME	4.056234837	
SiRNA-GSDME	18.59470367	14.19
SiRNA-GSDME	19.92995834	

Housekeeping gene (B2M)		
**simple**	**Cт value**	**Cт mean**
Control	15.26173401	
Control	14.72760868	14.93
Control	14.79530048	
Cisplatin	14.64215755	
Cisplatin	14.17390728	14.51
Cisplatin	14.71698093	
Z-DEVD-FMK	16.07919502	
Z-DEVD-FMK	15.38218117	15.91
Z-DEVD-FMK	16.26582146	
SiRNA-GSDME	15.38757133	
SiRNA-GSDME	14.97381496	15.43
SiRNA-GSDME	15.93342209	
Control	13.60147858	
Control	14.00483036	14.18
Control	14.94456387	
Cisplatin	12.93541813	
Cisplatin	15.62017345	14.13
Cisplatin	13.8371582	
Z-DEVD-FMK	16.54419136	
Z-DEVD-FMK	15.01327991	16.06
Z-DEVD-FMK	16.62574005	
SiRNA-GSDME	16.22640419	
SiRNA-GSDME	14.96789265	11.79
SiRNA-GSDME	4.178934574	

Housekeeping gene (SDHA)

simple	Cт value	Cт mean
Control	18.00235367	
Control	17.98171806	18.01
Control	18.05559349	
Cisplatin	17.87636757	
Cisplatin	20.27271652	18.63
Cisplatin	17.74117661	
Z-DEVD-FMK	19.87063789	
Z-DEVD-FMK	18.69654846	19.59
Z-DEVD-FMK	20.21102333	
SiRNA-GSDME	18.96853638	
SiRNA-GSDME	17.58195496	18.30
SiRNA-GSDME	18.36189461	
Control	17.95265388	
Control	17.7766552	17.93
Control	18.04955101	
Cisplatin	17.80585098	
Cisplatin	20.29578781	18.66
Cisplatin	17.87076378	
Z-DEVD-FMK	19.94425011	
Z-DEVD-FMK	18.75858116	19.61
Z-DEVD-FMK	20.12278557	
SiRNA-GSDME	18.93782997	
SiRNA-GSDME	17.92501831	18.56
SiRNA-GSDME	18.81580544	

Housekeeping gene (RPLP0)		
**simple**	**Cт value**	**Cт mean**
Control	11.67945004	11.74

Control	11.54940033	
Control	11.97746086	
Cisplatin	11.77974319	
Cisplatin	14.25026703	12.56
Cisplatin	11.65986252	
Z-DEVD-FMK	15.24375534	
Z-DEVD-FMK	13.67670631	14.38
Z-DEVD-FMK	14.23427391	
SiRNA-GSDME	13.26019764	
SiRNA-GSDME	11.89381123	12.51
SiRNA-GSDME	12.3776722	
Control	11.62778473	
Control	11.55079269	11.68
Control	11.85544586	
Cisplatin	11.60196972	
Cisplatin	14.16727448	12.44
Cisplatin	11.53700161	
Z-DEVD-FMK	15.96361828	
Z-DEVD-FMK	12.663414	14.33
Z-DEVD-FMK	14.3496933	
SiRNA-GSDME	13.21180534	
SiRNA-GSDME	12.04097652	12.63
SiRNA-GSDME	12.64858532	
